# Simultaneous Processing of Noun Cue and to-be-Produced Verb in Verb Generation Task: Electromagnetic Evidence

**DOI:** 10.3389/fnhum.2017.00279

**Published:** 2017-05-30

**Authors:** Anna V. Butorina, Anna A. Pavlova, Anastasia Y. Nikolaeva, Andrey O. Prokofyev, Denis P. Bondarev, Tatiana A. Stroganova

**Affiliations:** ^1^MEG Center, Moscow State University of Psychology and EducationMoscow, Russia; ^2^National Research Center “Kurchatov Institute”Moscow, Russia

**Keywords:** association, semantic retrieval, lexical–semantic processing, verb generation, word production, magnetoencephalography (MEG)

## Abstract

A long-standing but implicit assumption is that words strongly associated with a presented cue are automatically activated in the memory through rapid spread of activation within brain semantic networks. The current study was aimed to provide direct evidence of such rapid access to words’ semantic representations and to investigate its neural sources using magnetoencephalography (MEG) and distributed source localization technique. Thirty-three neurotypical subjects underwent the MEG recording during verb generation task, which was to produce verbs related to the presented noun cues. Brain responses evoked by the noun cues were examined while manipulating the strength of association between the noun and the potential verb responses. The strong vs. weak noun-verb association led to a greater noun-related neural response at 250–400 ms after cue onset, and faster verb production. The cortical sources of the differential response were localized in left temporal pole, previously implicated in semantic access, and left ventrolateral prefrontal cortex (VLPFC), thought to subserve controlled semantic retrieval. The strength of the left VLPFC’s response to the nouns with strong verb associates was positively correlated to the speed of verbs production. Our findings empirically validate the theoretical expectation that in case of a strongly connected noun-verb pair, successful access to target verb representation may occur already at the stage of lexico-semantic analysis of the presented noun. Moreover, the MEG results suggest that contrary to the previous conclusion derived from fMRI studies left VLPFC supports selection of the target verb representations, even if they were retrieved from semantic memory rapidly and effortlessly. The discordance between MEG and fMRI findings in verb generation task may stem from different modes of neural activation captured by phase-locked activity in MEG and slow changes of blood-oxygen-level-dependent (BOLD) signal in fMRI.

## Introduction

The retrieval of an intended word from memory storage is a crucial process for speech production. An average speaker knows about 30,000 lexical entries, yet, is capable to pick out an appropriate word less than in a half a second (Levelt, 1993).

According to conventional models, such high speed of word retrieval is based on well-learned word associations, which become embedded in the structure of semantic memory through frequent co-occurrence in the experience (Ferrer i Cancho and Solé, 2001; Nelson et al., 2004). The network of the links between the stored word representations is supposed to reflect meaningful relations between the corresponding words (Collins and Quillian, 1969; Collins and Loftus, 1975; Anderson, 2000; Steyvers and Tenenbaum, 2005). The links can vary in strength depending on similarity in meaning and/or frequency of co-occurrence: the closer the relationship between words is, the stronger the link is. The key premise for many word production studies lies in that strong links enable quick and automatic retrieval of the related words (e.g., Badre and Wagner, 2002; Whitney et al., 2011). Putatively, activation can spread across the strong links from one representation to another automatically, i.e., without additional effort or time delay.

Most of the evidence for spreading-activation retrieval comes from word perception studies, particularly, from the studies of subliminal semantic priming. When a target word (e.g., sugar) is preceded by a masked or short-presented (<80 ms) semantically related prime word (e.g., salt), the responses to the target tend to be shorter and more accurate, in comparison with a situation when the prime is semantically unrelated (e.g., cat; for a review, see Holcomb, 1988). It is generally presumed that once the prime representation has been activated, activation spreads across the links to the related nodes but does not reach the detached representations of unrelated words. Thus, when the related target is presented, its representation is already partially activated and requires less processing to reach a recognition threshold. Considering that subliminal presentation precludes strategic use of the prime as a cue for semantic retrieval, it appears that activation spreads across the network passively, in a mechanistic-like fashion (Neely, 1991; Neely and Kahan, 2001).

The electrophysiological studies of semantic priming show that the prime-target relation modulates neural processing of the target at 300–500 ms after its presentation. The amplitude of the N400 component of the event related potential (ERP) is reduced (more positive), when the prime and the target are semantically related (for review see Kutas and Federmeier, 2011). In general, the amplitude of the N400 response is regarded as reflecting the “ease” of access to stored semantic representations: the higher the amplitude is, the more effort is required (Kutas and Federmeier, 2000; Lau et al., 2008). The reduced N400 amplitudes were suggested to be associated with a facilitated access to target representation when the prime can pre-activate the target.

According to the word production studies (e.g., free association task, noun and verb generation task, etc.), the prime word acts as a cue, which not only facilitates but also triggers a reactivation of a related word. It has been assumed that the same spreading activation mechanism provides the automatic retrieval of representations, strongly connected to the cue word (e.g., Badre and Wagner, 2002; Playfoot et al., 2016). Once the cue representation has been activated in the memory, activation spreads to related nodes in accordance with the strength of the connections. The representations connected via strong links will receive stronger activation than those with weak connections and, therefore, can quickly reach the level of activation required to be chosen for production.

This assumption has been implicitly adopted by numerous fMRI word production studies that used the generation of strongly associated words as a “default” condition to examine how the production mechanism copes with effortful retrieval. Thus, in the milestone article of Thompson-Schill et al. (1997) participants were asked to name a related verb in response to the presented noun cue. Verbal responses were slower and fMRI activation was greater when nouns were associated with many verbs (e.g., the noun “map” is associated with verbs like “travel”, “find”, “draw” etc.), compared to the nouns with one dominant response option (e.g., the noun “apple” is strongly associated with the verb “eat”). Specifically, greater activation of left ventrolateral prefrontal cortex (VLPFC) was systematically observed in condition with multiple response options that put greater demand on retrieval control (Thompson-Schill et al., 1997; Barch et al., 2000; Persson et al., 2004; Snyder and Munakata, 2008; Crescentini et al., 2010; Snyder et al., 2011). It has been argued that in the absence of strong univocal noun-verb association, automatic stimulus-driven access to the target word is unsuccessful, and top-down control from the prefrontal regions is required to perform effortful search in memory (e.g., Wagner et al., 2001; Badre and Wagner, 2002, 2007; Martin and Cheng, 2006).

This interpretation omits neural mechanisms that carry out fast and effortless retrieval of the target verb. Surprisingly, neural basis of automatic access to target representation, although being a cornerstone of theoretical language modeling, is largely overlooked in the neuroimaging research. This gap in our understanding can be partially attributed to low temporal resolution of fMRI, as it is ill-suited for capturing transient neural processes associated with rapid automatic word retrieval. This limitation could be resolved by adopting magnetoencephalography (MEG), which allows brain activation tracking up to millisecond temporal precision and has reasonable spatial accuracy.

Here, using MEG, our aim was to explicitly test, whether the automatic access to response’s semantic representation is indeed as early process as the retrieval-through-spreading-activation hypothesis suggests. Critical prediction from this account is that the retrieval of the words strongly associated with the presented cue can occur simultaneously with the cue’s semantic analysis due to the activation spreading within the semantic networks (e.g., Badre and Wagner, 2002).

To test this hypothesis we utilized verb generation task, where subjects overtly named an action associated with visually presented noun. In one experimental condition, the cues consisted of nouns with one strongly associated verb; in another case, the noun cues were weakly associated with many appropriate verbs without any clearly dominant option. We expected that strong noun-verb association would modulate event-related fields (ERFs, the magnetoencephalographic equivalent of ERPs), elicited by the noun cue. If the ease of verb production affects brain response to the noun during time window associated with its semantic processing, it will be strong evidence that the automatic target word retrieval is coupled with the cue’s semantic processing.

## Materials and Methods

### Participants

Thirty-five volunteers (age range 20–48, mean age 26, 16 females) underwent MEG recording. All participants were native Russian-speakers, right-handed, had normal or corrected-to-normal vision and reported no neurological diseases or dyslexia. One subject was subsequently excluded from the analysis due to insufficient quantity of correct responses and another one due to MEG acquisition error. The final sample comprised 33 subjects. This study was carried out in accordance with the recommendations of The American Psychological Association’s (APA) Ethical Principles of Psychologists and Code of Conduct with written informed consent obtained from all subjects. The study was approved by the Ethics Committee of the Moscow State University of Psychology and Education.

### Materials

Sixty-five Russian nouns strongly associated with single verb and 65 nouns without univocal noun-verb association were selected as the production cues for overt verb generation task during an independent norming study. Four-hundred and nine nouns were taken from the frequency dictionary of modern Russian language (Lyashevskaya and Sharov, 2009), based on the concreteness of the word, (confirmed by the Russian National Corpus^1^), and its length, (between 4 and 10 letters). Fourty native Russian subjects (20–40 years old, 18 females), were presented with the nouns in random order and asked a question: “What this noun does?”. The task required naming an associated verb in the inflected form as if to form a short sentence (e.g., “solntse—svetit/sun—shines”). Later the verb responses were used to distinguish the nouns with one dominant verb associate from the ones with many weakly associated verbs. The association strength measure, i.e., a proportion of the participants who generated the same verb to each noun, was calculated (Martin and Cheng, 2006). If the majority of the participants (from 58% to 90%) responded with the same verb to the presented noun, it was considered to have one dominant verb associate and was included into the Strong Association (SA) condition (e.g., “solovey—poyet/nightingale—sings”). If less than 23% of the norming sample agreed on the same response, the noun was assigned to the Weak Association (WA) condition (e.g., “bumaga—mnetsya, goryt, rvetsya/paper—crumples, burns, tears”). As a result, two lists of 65 nouns each were compiled for the verb generation task (see the SA and WA nouns in Supplementary Material Appendix A1).

The mean word length, form frequency and number of lexical associates were assured to be similar between both lists (Table 1). Word form frequency was taken from Lyashevskaya and Sharov (2009) frequency dictionary. A number of lexical associates were taken from Russian Associative Thesaurus (Karaulov et al., 2002). The resulting items from SA and WA categories did not differ in average word length (*F*_(1,64)_ = 0.98, *p* = 0.52), word form frequency (*F*_(1,64)_ = 1.63, *p* = 0.02 with p-level equal to 0.01) and number of lexical associates (*F*_(1,64)_ = 0.77, *p* = 0.38).

**Table 1:** Means (and standard deviations) for psycholinguistic parameters of the nouns from Strong and Weak Association categories.

	Strong Association	Weak Association
Length in letters	5.6 (±1.6)	5.7 (±1.45)
Word form frequency (occurrence per million)	50.6 (±87.3)	49.1 (±65.8)
Number of lexical associates	72.9 (±52.6)	85.6 (±59)

### Design and Procedure

The participants were visually presented with the noun cues divided into 14 blocks of eight nouns each and two blocks comprised of nine nouns. The blocks contained either SA or WA cues, randomized within a block. Stimuli were written in a white font on a black background and presented on a screen placed at 1.5 m in front of the participant. The size of the stimuli did not exceed 5° of visual angle. The experiment was implemented in the Presentation software (Neurobehavioral Systems, Inc., Albany, CA, USA).

**Figure 1:**
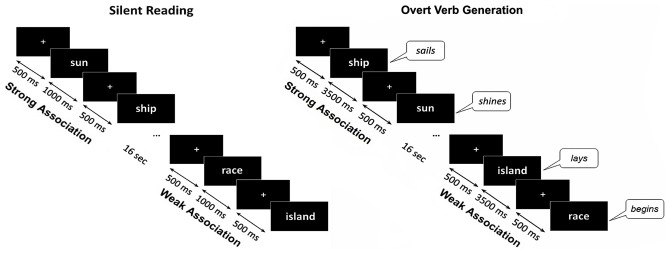
Schematic description of the experimental design. The same noun cues were visually presented during silent reading and overt verb generation tasks. The nouns from Strong Association and Weak Association categories were presented within blocks of eight or nine nouns, one category per block, alternating throughout each task. During the verb generation task the cues were presented for 3500 ms with a 300–500 ms interstimulus interval. During the reading task the noun cues were presented for 1000 ms. During Verb Generation task the subjects were required to verbally respond to the presented noun with an associated verb. The verbal responses were tape recorded and responses’ onsets were detected by an accelerometer placed on the participant’s throat.

Each noun was presented within two different experimental sessions. Within the control session (silent reading) the participant’s task was to read words inwardly. The trials began with presentation of a white fixation cross that randomly varied in duration for 300–500 ms, followed by the noun cue which remained on the screen for 1000 ms. Within the main session (verb generation) a participant was required to produce the verb associated with a presented noun by answering a question: “What this noun does?”. The instruction implied verb inflection and, if necessary, modification of the verb into a reflexive form. During the verb generation task each noun appeared on the screen for 3500 ms and was preceded by the white fixation cross presented for 300–500 ms. Nouns blocks from SA and WA categories were alternated with 16 s interval (Figure 1).

In course of verb generation session, participants’ vocal responses were tape recorded and checked for response errors. The trials with no or semantically unrelated responses, incomprehensible verbalizations, imprecise vocalization onsets, and with pre-stimulus intervals overlapping with the vocal response to the previous stimulus were excluded from subsequent analysis. As the verb responses were to be modified in person, number and form, we considered, although semantically correct, but erroneously modified verbs (e.g., “kvartyra—ubirayet/apartment—cleans” instead “kvartyra—ubirayetsya/apartment—is cleaned”), as errors and removed them from reaction time calculations and further MEG analysis. Overall, 2.6% (±3.4%) of responses in SA condition and 17.8% (±8.5%) in WA were excluded.

The reaction time of the each verb production was calculated based on the measures provided by three-axis accelerometer located on the participant’s throat (ADXL330 iMEMS Accelerometer, Analog Devices, Norwood, MA, USA). Speech onsets were marked using an automated algorithm (Zakharova et al., 2012), that detected increases in the accelerometer signal (*z* axis) above baseline by three standard deviations, reaching peak amplitude (2.5× threshold) within a 3500 ms time window, and then visually inspected for false positives. The resulting reaction times and error rates were subjected to the analysis of variance (ANOVA).

### MEG Data Acquisition

MEG data were acquired inside a magnetically shielded room (AK3b, Vacuumschmelze GmbH, Hanau, Germany), using a dc-SQUID Neuromag^TM^ Vector View system (Elekta-Neuromag, Helsinki, Finland) with 204 planar gradiometers and 102 magnetometers. Data were sampled at 1000 Hz and filtered with a band-passed 0.03–333 Hz filter. The participants’ head shapes were measured by a 3Space Isotrack II System (Fastrak Polhemus, Colchester, VA, USA) by digitizing three anatomical landmark points (nasion, left and right preauricular points) and additional randomly distributed points on the scalp. While recording, the position and orientation of the head were monitored by four Head Position Indicator coils. The electrooculogram was registered with two pairs of electrodes located above and below the left eye and at the outer canthi of both eyes for recording of vertical and horizontal eye movements respectively. MRI scans were acquired for 28 participants with a 1.5 T Philips Intera system and were used for reconstruction of the cortical surface using Freesurfer software^2^. Due to the MRI acquisition error, the rest five participants’ head models have failed to be obtained.

### MEG Pre-Processing

The raw data were subjected to the temporal signal space separation (tSSS) method (Taulu et al., 2005), embedded in MaxFilter program (Elekta Neuromag software), aimed to suppress magnetic interference coming from sources distant to the sensor array. Biological artifacts (cardiac fields, eye movements, myogenic activity), were corrected using the SSP algorithm embedded in Brainstorm software (Tadel et al., 2011). To compensate for the within-block head-movement (as measured by Head Position Indicator coils) a movement compensation procedure was applied. For sensor-space analysis, the data were converted to standard head position (*x* = 0 mm; *y* = 0 mm; *z* = 45 mm) across all blocks.

Data were divided into epochs of 1300 ms, from 300 ms before to 1000 ms after stimulus onset. The baseline correction was computed using the interval from the −300 ms to −50 ms before the stimulus onset. Epochs were rejected if the peak-to-peak value over the epoch exceeds 3 × 10-10 T/m (gradiometers) and 12 × 10-10 T/m (magnetometers) channels. Average number of verb generation trials finally taken for the analysis was 63 ± 2 in SA condition and 53 ± 5 in WA (*t*_(32)_ = 11.22, *p* < 0.001).

### MEG Data Analysis

The analysis of verb generation data had two steps. In order to describe the general time course of brain response to the noun cue, we merged the MEG data across both conditions and compared it with the baseline activity. This was followed by a source analysis intended to characterize the spatial-temporal cortical dynamics underlying visual noun cue processing in verb generation task. The second part of the analysis was aimed to identify putative effect of noun-verb association strength on neural activity evoked by noun cue presentation, both in terms of its timing and involved brain regions. To ensure that association strength was the main factor affecting the brain responses to the nouns from SA and WA categories we checked for SA-WA differences in the silent reading task.

### Analysis of Phase-Locked Response to a Noun Cue

The analysis devoted to general spatio-temporal dynamics of noun cue processing was conducted using Brainstorm software (Tadel et al., 2011). Only those 28 participants whose MRI scans were obtained entered the source analysis. The individual ERFs were computed by pooling together the trials from both conditions and averaging them over a 300 ms prestimulus interval and a 1000 ms post-stimulus for each of the 306 sensors. The cortical sources of the evoked responses were modeled by a “depth-weighted” linear L2-minimum norm estimation method (Hämäläinen and Ilmoniemi, 1994). The individual cortical surfaces were imported from FreeSurfer and tessellated with 15,000 nodes. The forward solution was calculated using overlapping spheres approach (Huang et al., 1999). The inverse solution was computed by Brainstorm built-in minimum norm estimation algorithm applied with the default settings (“kernel only” as the output mode, three as the signal-to-noise ratio, the source orientation constrained to perpendicular to the cortical surface, the depth weighting restricting source locations to the cortical surface and the whitening PCA). A noise covariance matrix, necessary to control noise effects on the solution (Bouhamidi and Jbilou, 2007), was calculated over −300 to −50 baseline interval (Dale et al., 2000).

The individual source maps were projected to the cortical surface of the Montreal Neurological Institute brain template (MNI-Colin27) and a grand-average was computed. Differences in source activation between participants’ responses and baseline period were tested via paired *t*-tests under significance level of *p* < 0.01 (FDR-corrected with Brainstorm built-in algorithm).

### Analysis of SA-WA Differences in the Noun-Evoked Response

The difference in the magnitude of noun-evoked response in SA vs. WA was examined using Statistical Parametric Mapping software (SPM12: Wellcome Trust Centre for Neuroimaging, London^3^). For analysis of evoked magnetic fields the planar gradiometers data were converted to a Matlab-based, SPM format, epoched −300 ms to 1000 ms around stimulus onset, and baseline corrected over −300 to −50 prestimulus interval. The epoched data were averaged separately across each condition, using a SPM built-in robust averaging procedure (Holland and Welsch, 1977). For each gradiometer pair data were combined by calculating the root-mean-square values. The resulting 3D files of space (32 × 32 pixels) and time (1500 ms) dimensions were converted to images of Neuroimaging Informatics Technology Initiative (NIfTI) format.

For statistical analysis the topography × time images were smoothed in space-time using a Gaussian smoothing kernel with Full Width Half Maximum of 8 mm × 8 mm × 8 ms to ensure that the images conform to the assumptions of Random Field Theory (Kilner and Friston, 2010). Then, the smoothed images from SA and WA conditions were subjected to a paired *t*-test with uncorrected threshold of *p* < 0.0001. The resulting spatial-temporal clusters of significant SA-WA differences underwent the family-wise error rate (FWE) correction with cluster-level threshold of *p* < 0.05. Only the clusters that survived cluster-level correction were used to guide the subsequent analysis in the source space.

Source reconstructions of evoked magnetic fields were created by the means of Multiple Sparse Priors, the Bayesian source inversion algorithm of SPM12 (Friston et al., 2008). To determine the cortical areas that contribute to the significant SA-WA differences in the brain response, we used the sensor-level spatial-temporal clusters as the mask defining the time windows of interest and a broad cortical region of interest for source-level analysis. Within the time windows of interest the individual source maps were averaged across all time points in each experimental condition separately, then overlaid with the spatial mask and subjected to between-condition paired *t*-test. Given that the source-space analysis was guided by FWE-corrected sensor-level results, the statistical threshold in the source-space was defined at *p* < 0.05 (peak-level, uncorrected). We reconstructed the time courses at the peak vertex for each target cluster.

## Results

### Behavioral Results

As expected, verb generation was faster and more accurate when the noun cues had strong verb associations (*M* = 1.22 ± 0.17 s, 1.76 ± 2.28% errors) compared to the nouns with weakly associated verbs (*M* = 1.89 ± 0.23 s, 11.76 ± 5.51% errors). ANOVA revealed large and highly significant effect of association strength on error rate (*F*_(1,32)_ = 167.6, *p* < 0.0001; *η*^2^ =0.84) and reaction times (*F*_(1,32)_ = 325; *p* < 0.0001; *η*^2^ = 0.91).

### Electrophysiological Results

#### Event-Related Response to the Visual Noun Cue in Verb Generation Task

##### Sensor-level clusters

**Figure 2:**
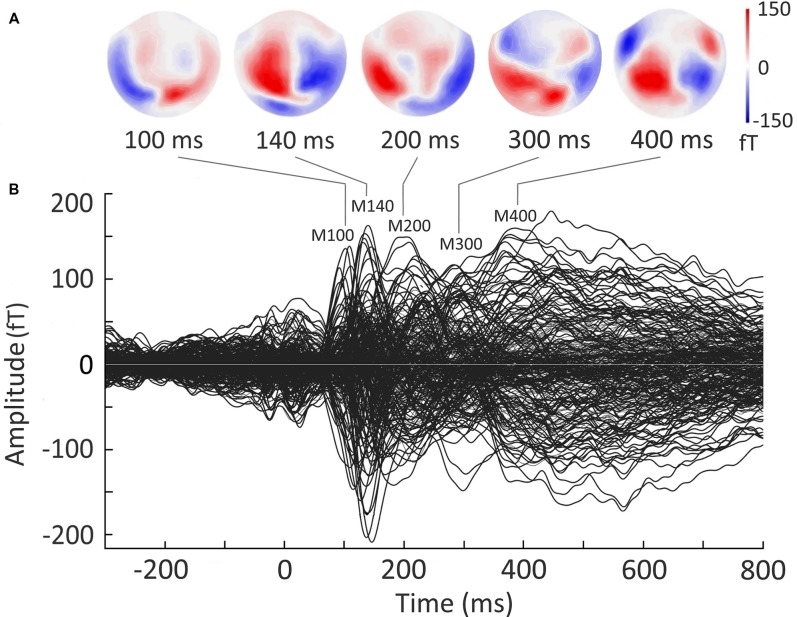
Grand average sensor-level magnetoencephalography (MEG) evoked response to visually presented nouns in verb generation task. Responses to the nouns from Strong Association and Weak Association categories are pooled together. Strength of magnetic fields is represented in femto-Tesla (fT). **(A)** Field distribution of the evoked response at the time points derived from the peaks in the butterfly plot. **(B)** Butterfly plot of MEG evoked waveforms from all MEG channels. Zero point denotes the onset of the noun cue. The increase of the response around zero is related to the fixation cross presentation.

Figure 2B presents a butterfly plot of overlapped evoked responses from all MEG channels averaged across both experimental conditions. The noun cue presentation elicited the evoked response with characteristic narrow peaks around 100 and 140 ms after cue onset, followed by broader components around 200 and 400 ms. Two early components (at 100 ms and 140 ms) demonstrated typical posterior scalp distributions (Figure 2A) that allowed to identify them as MEG counterparts of P100 and N170 components established in visual word recognition ERP studies (e.g., Hauk and Pulvermüller, 2004; Maurer et al., 2005). The response at 200–300 ms corresponded to the time window of N250 component (e.g., Holcomb and Grainger, 2006), and showed bilateral temporoparietal distribution. In the time-window of  “semantic” N400 component (300–450 ms; e.g., Lau et al., 2008; Kutas and Federmeier, 2011), the response shifted to the temporo-frontal scalp regions.

##### Source-level analysis

**Figure 3:**
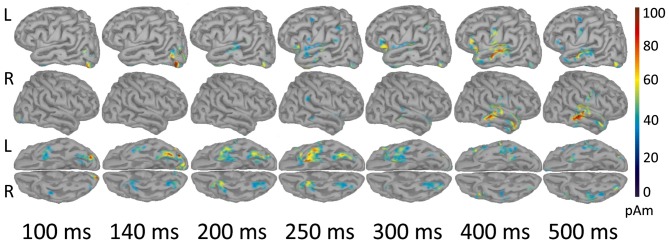
Reconstructed temporal sequence of cortical activations for the noun-evoked response in verb generation task. The strength of cortical sources is shown at the time points corresponding to the peaks of event-related fields (ERFs) components. Strong and Weak Association conditions are pooled together. Only the significant clusters of activation as compared to baseline are shown (*p* < 0.01, FDR-corrected). Colorbar represents response strength in picoamperes (pAM). Note, that cortical response evoked by visually presented noun cue progressed along the posterior-anterior axis from posterior sensory regions to more anterior multimodal association areas.

As shown in Figure 3, early components of the evoked response (M100) were localized to occipital cortex bilaterally. At 140 ms the response became more left-lateralized and shifted to the inferior occipitotemporal cortex, including the region of left fusiform gyrus. By 200 ms activation spread to more anterior brain regions and reached the anterior part of left temporal lobe (ATL). At 250–300 ms the response engaged part of the left VLPFC (left inferior frontal gyrus, Brodmann area 47 (BA 47)), and the areas close to the left auditory cortex, in the left transverse gyrus. After 400 ms the response centered at the cortex adjacent to the left superior temporal sulcus, and also comprised of the left VLPFC and the left inferior pericentral region. By 500 ms post-stimulus the peak of response shifted to the right middle and superior temporal gyri. MNI coordinates of the activation peaks at the respective time points (significant under *p* < 0.01, FDR-corrected at the cluster level), are presented in Table 2.

**Table 2:** Cortical sources of the components of the noun-evoked event-related fields (ERFs) in verb generation task.

Time points (ms)	Region	MNI coordinates			~BA
		*X*	*Y*	*Z*	
100	R. Striate cortex	13	−102	−6	BA 17
140	L. Inferior occipital gyrus	−37	−89	−12	BA 18
200	L. Fusiform gyrus	−36	−76	−17	BA 19
250	L. Inferior temporal area	−36	−2	−39	BA 20
300	L. Inferior frontal gyrus	−50	15	−6	BA 47
400	L. Middle temporal gyrus	−61	−15	−6	BA 21
500	R. Middle temporal gyrus	68	−32	−4	BA 21

*Time points corresponds to the peaks of ERFs components. Cortical coordinates in MNI space, Brodmann areas (BA) and anatomical specifications are given to the vertices with maximal source strength at the respective time points. The activation of all the sources is significantly greater than baseline (*p* < 0.01, FDR-corrected)*.

In our study, the spatiotemporal pattern of noun-related evoked activity was generally consistent with the current models of visual word recognition (e.g., Grainger and Holcomb, 2009; Carreiras et al., 2014). These models postulated a spread of activation along the posterior-anterior axis of the brain after visual word presentation. As the written word analysis progresses to higher-level features, the response shifts from posterior sensory regions to anterior multimodal association areas.

#### Effects of Noun-Verb Association Strength on Noun Cue Processing

##### Sensor-level analysis

**Figure 4:**
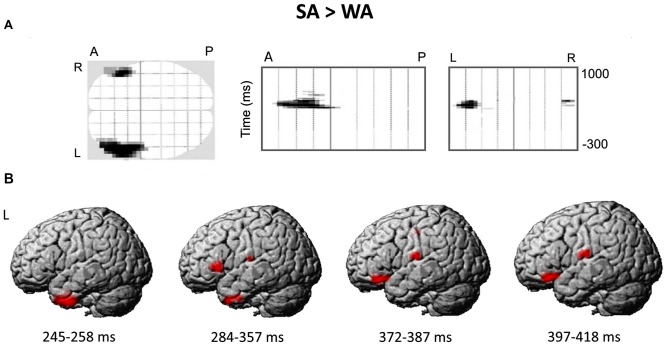
The differential brain response to the nouns from Strong Association (SA) and Weak Association (WA) categories: statistical parametric mapping analysis in sensor and source space. **(A)** The three projections (SPM glass image) show the sensor array from above (transverse), the right (sagittal) and the back (coronal). A-anterior, P-posterior, L-left and R-right parts of the array. Areas in black correspond to spatial clusters with significant sensor-level differences in ERF between the SA and WA nouns (*t*-test, *p* < 0.05, family-wise error rate (FWE)-corrected). All the clusters reflect greater response to the SA vs. WA nouns within four time windows of 245–258 ms, 284–357 ms, 372–384 ms and 397–418 ms after the cue onset, and are spatially confined to the left anterior quadrant of the sensor map. No clusters with opposite direction of the effect were found. **(B)** The reconstruction of cortical sources underlying greater ERF to the SA vs. WA nouns. Cortical sources were modeled within the time windows defined by the significant results of the sensor-level analysis. The statistical maps were threshold using a voxel-wise statistical threshold of *p* < 0.05.

Figure 4A presents the spatial-temporal clusters differentiating the responses to the noun cues with strong and weak verb associates in verb generation task. The largest and the most significant spatial-temporal cluster (cluster-level FWE correction *p* < 0.0001), was located over the anterolateral region of the left hemispheric sensor array within the time interval of 284–357 ms after the noun cue onset. Other significant anterior left-hemispheric clusters emerged either earlier—at 245–258 ms (cluster-level FWE correction *p* < 0.002) or later at 372–384 and 397–418 ms (*p* < 0.05, FWE corrected) after stimulus onset. One right hemispheric cluster survived correction for multiple comparisons (*p* < 0.05, FWE corrected): it appeared at 408 ms and was located in the anterior part of the right hemispheric sensor array. Notably, for all the significant clusters the noun-evoked response was stronger to the nouns from SA category as compared to WA one. No spatial-temporal clusters demonstrating the opposite direction of the association strength effect were found.

During silent reading task the ERFs to the nouns from SA and WA categories did not differ and both contrasts produced no significant SPM clusters. Therefore, we concluded that differences found between brain responses to SA and WA nouns in verb generation task resulted from specific task demands for retrieval of an associated verb.

##### Source-level analysis

Figure 4B shows the reconstructed cortical sources of significant SA-WA difference in ERFs within the response time windows specified above. Since significant effect of association strength at the gradiometer level was mostly confined to the left anterior quadrant of the sensor array, we considered cortical locations for source-space reconstruction only within left frontal lobe and ATL.

The earliest effect of association strength was observed within 245–258 ms time window and was localized to the left ATL (peak level *p*-value = 0.009). Within the next time interval (284–357 ms), in addition to the left ATL (*p* = 0.023), the effect comprised the left VLPFC (BA 45/47; *p* = 0.014) and the left transverse temporal gyrus (*p* = 0.024). Further on, at 372–387 and 397–418 ms time windows the significant SA-WA differences in the response strength to the noun cue were concentrated in the left VLPFC (BA 47; *p* = 0.008 and *p* = 0.004, respectively) and the left postcentral gyrus (*p* = 0.007 and *p* = 0.014, respectively). Table 3 lists the MNI coordinates of peak vertices of the cortical sources modulated by noun-verb association strength.

**Table 3:** Cortical clusters demonstrating Strong Association vs. Weak Association contrast.

Time windows (ms)	Region	MNI coordinates			~BA	*p*-values (uncorrected)
		*X*	*Y*	*Z*		
245–258	L. Anterior superior temporal gyrus	−26	8	−36	BA 38	0.009
284–357	L. Anterior middle temporal gyrus	−36	6	−38	BA 38	0.023
	L. Inferior frontal gyrus	−52	22	0	BA 47	0.014
	L. Transverse temporal gyrus	−62	−16	14	BA 42	0.024
372–387	L. Inferior frontal gyrus	−42	32	−16	BA 47	0.008
	L. Postcentral gyrus	−64	−16	16	BA 43	0.007
	L. Postcentral gyrus	−50	−16	46	BA 3	0.042
397–418	L. Inferior frontal gyrus	−44	30	−16	BA 47	0.004
	L. Postcentral gyrus	−64	−14	18	BA 43	0.014

*Time windows of interest, anatomical specifications, MNI coordinates, Brodmann areas (BA) and *p*-values (peak-level) displayed for clusters with higher activation for nouns with strong vs. weak verb associates*.

**Figure 5:**
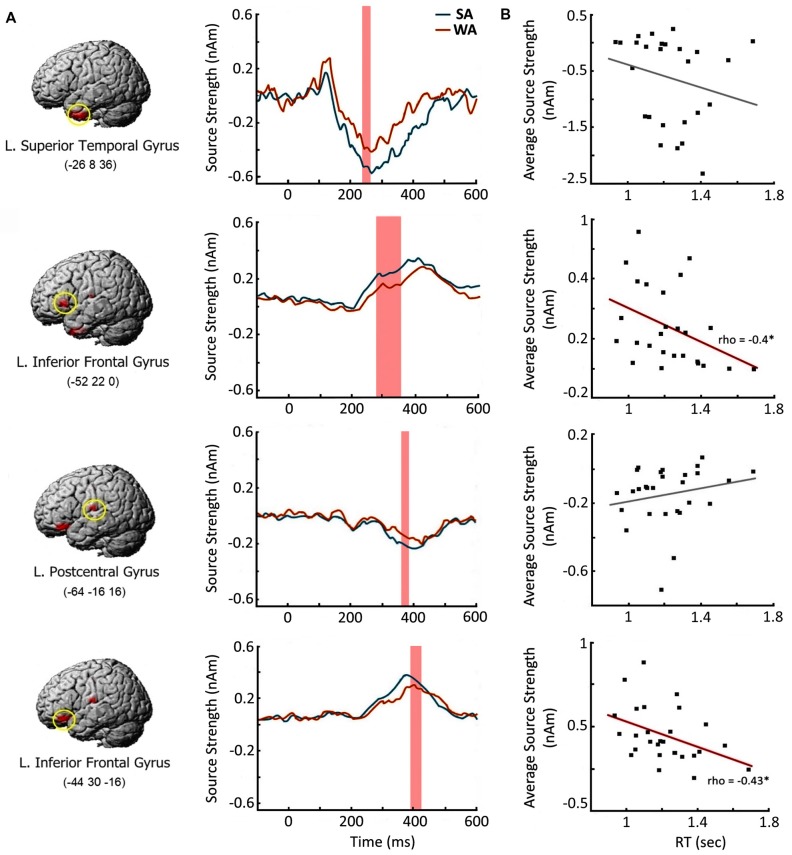
Brain response elicited by the Strong Association nouns: time course and relation to the speed of verb generation. **(A)** Cortical regions with greater response to Strong Association (SA) vs. Weak Association (WA) nouns and their time courses. Left column contains the brain images and their MNI coordinates. Middle column depicts the time courses in the peak vertex of the respective cortical region. Shaded bars denote the time windows with significant SA-WA differences. **(B)** Correlations between the activation strength in response to the noun cue and the speed of verb generation in SA condition. *X* axis denotes the reaction times in verb generation task in seconds (sec). *Y* axis represents the average source strength in nanoamperes (nAm) within the time windows of significant SA-WA differences. The significant Spearman correlations are marked by asterisks. Note, that greater cortical response to the noun cue in the left ventrolateral prefrontal cortex (VLPFC) is associated with the faster verb production.

Next, we examined whether the enhanced response to the nouns from SA category at the cortical regions specified above is beneficial for verbal production of the target verbs. We chose four cortical regions which demonstrated the most reliable effect of noun-verb association strength in each time window of interest (Table 3). For each region we reconstructed the SA activation time courses at the peak vertices for each participant (Figure 5A), The resulting source intensities were averaged across the respective time intervals and subjected to correlation analysis: computation of Spearman’s correlation between this brain measure and the speed of subject’s verbal response in SA condition. The response strength of both regions of the left VLPFC moderately correlated with the speed of verb generation (rho = −0.4, *p* = 0.04 and rho = −0.43, *p* = 0.03, respectively). Faster verb production was associated with greater evoked response (Figure 5B). Activation strength at two other regions was not related to subjects’ reaction time.

## Discussion

The present study examined neural processes underlying automatic semantic retrieval in word production. By measuring MEG from participants involved in verb generation task, we examined whether neural responses evoked by noun cues were modulated by the association strength between a cue and to-be-produced verb.

Our main finding is that the strength of noun-verb association affects noun’s processing already at 250–400 ms after stimulus onset, i.e., at the stage of semantic M400 component of noun-evoked response or even earlier. Thus, the retrieval of target verb does not necessarily require the noun’s processing to be completed but partially overlaps and interacts with its semantic analysis. This early interactive processing engages the left hemispheric regions of temporal pole and the VLPFC that are thought to be involved into semantic access, and also modulates the activity of left auditory area and the premotor cortex. Notably, the greater activation of all these regions characterizes the brain response to the nouns, which prompt fast and effortless generation of the related verbs, i.e., the nouns with one strong verb associate in comparison with those with many weakly associated verbs. Moreover, faster speed of verb production for such nouns is directly linked to the stronger activation they elicit in the regions of the left VLPFC. Prima facie, our findings contradict the well-known fMRI results that consistently show the exactly opposite effect: greater activation of the left VLPFC for more demanding retrieval of weakly associated verb (e.g., Thompson-Schill et al., 1997; Barch et al., 2000; Snyder and Munakata, 2008; Crescentini et al., 2010; Snyder et al., 2011). Further in the discussion we will address the functional meaning of our findings as well as the seemingly conflicting results observed in MEG and fMRI studies of verb generation task. It will be done from the perspective of current views on the neural origin of speech-related evoked responses and their relations to automatic retrieval of written word semantics.

### Recognition Process of Visually Presented Noun Cue

In our study, the earliest response elicited by the visually presented noun cue was observed at approximately 100 ms in the bilateral occipital cortex and, presumably, was associated with early visual analysis of sub-letter features (Tarkiainen et al., 1999). At 140–200 ms the response maximum shifted to the inferior posterior occipito-temporal cortex. The existing MEG and fMRI literature agrees that these region, (more specifically the left fusiform gyrus), is related to matching high-level orthographic representation with lexical information (Nobre et al., 1994; Tarkiainen et al., 1999, 2002; Dehaene et al., 2002; Dehaene and Cohen, 2011).

Further on, at 200 ms post-stimulus an activation spread to the ATL and was sustained there until 500 ms after the word onset (Figures 3, 5), the timing that is closely coincides with the reported in the previous MEG, ECoG and TMS literature (McCarthy et al., 1995; Halgren et al., 2002; Lau et al., 2013; Jackson et al., 2015). There is mounting evidence implicating the ATL in semantic processing as an amodal hub, which mediates and integrates between modality-specific word representations distributed over the cortex (Rogers et al., 2004; Patterson et al., 2007; Lambon Ralph et al., 2010; Lambon Ralph, 2014). According to our research, the left ATL activation at 200 ms post-stimulus could reflect its engagement into the process of activation of the stored supra-modal representations of a visually presented noun. In line with this suggestion, we observed that the ATL response to visual word was immediately followed by the activation of the auditory cortex and speech-related regions of the temporal lobe (Figure 3), which could reflect re-encoding of orthographic stimuli to its phonological representation (Haist et al., 2001; Sekiguchi et al., 2004).

The response at 300–500 ms, (the time window of “semantic” M400 component), engaged a number of spatially distributed cortical regions including the posterior middle temporal gyrus (pMTG) with adjacent region of the superior temporal gyrus/sulcus bilaterally, and the left VLPFC (Figure 3). The region around the superior temporal sulcus is traditionally referred to as a long-term storage of semantic representations (for meta-analysis, see Binder et al., 2009), whereas, the anterior-ventral part of the left VLPFC is suggested to be involved in a top-down control of the semantic retrieval that ensures access to the semantic features relevant to the current task (e.g., Thompson-Schill et al., 1997; Wagner et al., 2001; Badre and Wagner, 2007).

Thus, the observed neural activity underlying processing of the visually presented noun cue in the verb generation task complies with the general scheme of visual word recognition (for recent reviews, see Salmelin, 2007; Dien, 2009; Grainger and Holcomb, 2009; Pulvermüller et al., 2009; Carreiras et al., 2014). The processing starts from low-level analysis of visual features in the occipital cortex (~100 ms), then proceeds along the ventral visual stream to more complex analysis of word visual form in the left posterior occipitotemporal cortex (~140 ms) and culminates in sustained activity in the lexico-semantic areas of the left temporal and prefrontal cortex from around 200–250 ms onwards.

### Access to Verb Representation Is Coupled with the Processing of the Noun Cue

The nouns with strong and weak verb associates differ in the magnitude of the evoked brain response within 250–400 ms time window after the presentation. The nouns with strong verb associates followed by faster and more accurate verb generation, elicited greater brain response than the nouns with weakly associated verbs. Notably, this differential brain activation to the nouns from SA category was present only in the context of verb generation task but not during the nouns’ silent reading. Given that, we suggest the observed effects were caused by specific task requirements, i.e., to retrieve a verb, and not by task-irrelevant lexical variables that could potentially affect the brain responses to the SA and WA nouns.

Apart from lexical difference, greater attention allocation to the more complex task could be a confounding factor in the estimation of the relation between MEG event-related response to noun cue and noun-verb association strength. The generation of weakly associated verbs was more demanding, so the stimulus blocks of WA nouns could attract more attention than the blocks comprising the cues with strong prepotent responses. The attention effect on the amplitude of event-related responses is well-described in different experimental paradigms, including speech-related tasks with more attention-demanding condition provoking a relative increase of electric and magnetic evoked responses (e.g., McCarthy and Nobre, 1993; Ruz and Nobre, 2008) However, in our study, the evoked response was greater to the SA nouns, which were easier to respond to than WA nouns. That is why it is highly improbable that the effect of association strength on the noun-evoked response stemmed from attention demands.

Thus, the ease with which the target verb had been produced was the main factor influencing the processing of the noun cue. Significantly, the observed effect occurs at the time window of 250–400 ms after the cue onset suggesting the access to verbs’ representations is realized already at the stage of noun’s lexico-semantic processing. Conceivably, strong noun-verb association allows the activation from the cue representation to spread via the strong links to the representations of related verbs. At the same time, a WA prevents this fast access to the relevant representations devolving their retrieval to the later effortful processing mechanisms. Therefore, we can assume that in SA vs. WA condition, successful re-activation of noun-verb interconnected representation resulted in a larger amount of neural ensembles. Their combined response was reflected in a higher-amplitude noun-related ERFs.

Over the years, theoretical models of semantic networks have assumed that a cue leads to simultaneous activation of multiple interconnected representations (e.g., Collins and Loftus, 1975; Anderson, 2000). So, this automatic conjoint processing can explain fast and efficient production of the related words (e.g., Badre and Wagner, 2002). To the best of our knowledge, however, the current work is the first study that provides experimental evidence of co-activation of neural representations for nouns and verbs in verb generation task.

The activation automatically spreading between semantically related representations has been suggested as one of the mechanisms contributing to prediction in language processing (e.g., Van Petten, 1993; Federmeier and Kutas, 1999; Hoeks et al., 2004; Indefrey, 2011). Our findings prove that the cue word presented within a task-specific framework can (pre-) activate the neural representation of the strongly associated but not-encountered words based on well-learned relations in long-term memory, thus providing direct neural evidence for predictive coding theories.

The spatial-temporal pattern of differential neural responses to noun-cues strongly associated with their verbs is consistent with the putative simultaneous processing of noun-verb semantic representations. The earliest association strength effect on brain activation occurred at 250–350 ms in the left ATL, which may be responsible for a highly automatic activation of amodal semantic networks generally representing the noun with all its existing semantic associates (Fujimaki et al., 2009; Lau et al., 2013). In case of verb generation, the task may bias the re-activated links toward those connected to the verb associates. At this relatively early stage of conjoint noun-verb processing, the automatically activated verbs could be strongly associated but still task inappropriate, if they do not satisfy the task requirement to name the noun’s action. This may explain the absence of correlation between the magnitude of ATL activation and the speed of verb production in SA condition (Figure 5B).

Later on (280–420 ms post-cue), the conjoint noun-verb processing engages the left VLPFC representing the most anterior part of Broca’s complex (Hagoort, 2005). Given the general functional role of prefrontal cortex in the selection of task appropriate representation in underdetermined situations (e.g., Desimone and Duncan, 1995; Kan and Thompson-Schill, 2004), the left VLPFC’s involvement may be related to selective, goal-directed processing of successfully activated verb representations. The significant correlation found between the strength of the left VLPFC’s response and the speed of verb generation (Figure 5B) supports the idea that this region has a direct impact on the retrieval of the target word.

An alternative explanation of the VLPFC’s differential response might stem from other functions attributed to Broca’s complex such as phonological encoding and syllabification (e.g., Ghosh et al., 2008; Papoutsi et al., 2009; Indefrey, 2011). However, both relatively early timing of the observed differential response and its localization to the anterior part of inferior frontal gyrus refutes this interpretation. According to Indefrey (2011), the phonological encoding occurs not earlier than 255 ms before the response production. In our study, the association-strength-related modulation of the left VLPFC’s activity precedes the response for approximately 800 ms, thus, occurring long before the phonological encoding of the verb response is thought to begin. In spatial terms, phonological encoding has been shown to recruit posterior regions of inferior frontal gyrus (BA 44) and ventral part of BA 6, whereas association strength affected the activity within the most anterior part of inferior frontal gyrus—VLPFC/BA 47, previously implicated in semantic processing (Hagoort and Indefrey, 2014).

As we have mentioned above, the fMRI studies of verb generation (e.g., Thompson-Schill et al., 1997; Barch et al., 2000; Snyder and Munakata, 2008; Crescentini et al., 2010; Snyder et al., 2011) showed greater activation of the left VLPFC in WA compared to SA condition—the exactly opposite effect to the one reported here. We assume that the discordance between MEG/fMRI findings results from different mode of neural activation captured by changes in evoked, phase-locked response in MEG and blood-oxygen-level-dependent (BOLD) signal. Considering that the BOLD signal integrates brain hemodynamic changes over several seconds, fast and short-lived neural activation contributing to MEG/EEG evoked response could be difficult to detect with fMRI (Logothetis et al., 2001; Engell et al., 2012). In turn, ERF/ERP computation averages out the contribution of all non-stimulus-locked neural activity, which has been shown to correlate with changes in BOLD signal (Kayser et al., 2004; Koch et al., 2009; Sirotin and Das, 2009; Ojemann et al., 2010). The increasing evidence indicates that hemodynamic changes measured by fMRI are more compatible with non-phase-locked changes in EEG/MEG frequency power rather than with the phase-locked ERP/ERF (Engell et al., 2012; Singh, 2012).

These considerations lead to a conclusion that successful fast retrieval of task-appropriate verb is accompanied by the short-lived highly coherent neuronal activation of the left VLPFC, which is reflected in enhanced MEG phase-locked ERFs. A failure of automatic retrieval may trigger the effortful search for target representation in memory that manifests in the sustained long-lasting VLPFC’s activation captured by BOLD response. Thus, it is possible that the left VLPFC, contrary to common beliefs, supports selection of the target verb representations, even if they were retrieved from semantic memory rapidly and effortlessly.

The proposed shift from automatic to effortful retrieval in the left VLPFC remains highly speculative until it is not supported by the findings obtained within a single experimental design. In this study we restricted our analysis to the stimulus-locked neural response, thus highlighting “automatic” processing leading to fast and easy word production. We anticipate that the delayed and prolonged process of controlled verb retrieval will be reflected in sustained changes in the magnitude of neural oscillations, rather than in ERF source strength. This assumption leads to intriguing prediction of the opposite direction of SA-WA difference in “neural activation” depending on whether phase-locked or non-phase-locked MEG activity is chosen as an activation measure. In the latter case, the greater strength of MEG response should accompany the effortful retrieval of the verbs weakly associated with their nouns and should correlate with intensity of BOLD response. This hypothesis can be directly tested in the future research combining analysis of MEG phase-locked and non-phase-locked activity and hemodynamic response.

In conclusion, findings of the current study suggest that access to target verb representation in verb generation task co-occurs with lexico-semantic processing of the presented noun cue. To our knowledge, our results are among the first neuroimaging evidence supporting the idea that the target word representation may be retrieved through activation rapidly spreading along the strong links connecting a cue representation with its closest neighbors in semantic network (Badre and Wagner, 2002). Furthermore, the spatial-temporal pattern of brain activation implies that this seemingly automatic semantic retrieval of target verb engages the left VLPFC, which is known to be responsible for a task-relevant selection between available response alternatives.

## Author Contributions

AYN and AOP performed the experiment. AVB, AAP, DPB, AYN, AOP and TAS analyzed the data and interpreted the results. TAS and AAP wrote the manuscript. All the authors approved the final version of the manuscript.

## Conflict of Interest Statement

The authors declare that the research was conducted in the absence of any commercial or financial relationships that could be construed as a potential conflict of interest.

## Footnotes

^1^http://ruscorpora.ru/en

^2^http://surfer.nmr.mgh.harvard.edu/

^3^http://www.fil.ion.ucl.ac.uk/spm
