# Supplementary material for: Simultaneous Processing of Noun Cue and to-be-Produced Verb in Verb Generation Task: Electromagnetic Evidence
Source: Front Hum Neurosci. 2017 May 30;11:279. doi: 10.3389/fnhum.2017.00279 (PMC5447679; doi:10.3389/fnhum.2017.00279)
Supplement: Supplementary file 1 [file Data_Sheet_1.docx]

**Appendix**

*Experimental materials.* The noun cues for the two conditions in alphabetical order with their corresponding English translations.

| Strong Association | | Weak Association | |
| --- | --- | --- | --- |
| arbalet/crossbow | pianist/pianist | balkon/balcony | perila/railings |
| ballerina/ballet dancer | portnikha/dressmaker | banka/jar | peshchera/cave |
| bomba/bomb | povar/cook | brodyaga/tramp | plotina/dam |
| bronya/armor | printer/printer | bul'var/avenue | poddelka/fake |
| chaynik/kettel | reka/river | bumaga/paper | podokonnik/window sill |
| cheremukha/bird-cherry | ruchka/pen | chemodan/suitcase | polosa/ band |
| chervyak/worm | ruzh'ye/gun | chert/devil | poyas/belt |
| drel'/drill | samolet/plane | derevnya/village | puanty/ballet shoes |
| dukhi/perfume | shchepki/chips | devushka/young woman | pudra/powder |
| fen/hair dryer | shchetka/brush | fantik/wrapper | raduga/rainbow |
| garmon'/accordion | shchit/shield | iskra/spark | regalii/regalia |
| gorlo/throat | shlem/helmet | kamorka/ | rukav/sleeve |
| grom/thunder | shvabra/mop | karnaval/carnival | sapog/boot |
| kasha/porridge | skal'pel'/scalpel | khleb/bread | sherst'/wool |
| khor/chorus | sobaka/dog | kislota/acid | shov/seam |
| kley/glue | solntse/sun | korobka/box | skelet/skeleton |
| korabl'/ship | solovey/nightingale | kukla/doll | slon/elephant |
| koroleva/queen | stepler/stapler | kupal'nik/swim suit | sous/sauce |
| lastik/eraser | stol/table | kuzov/carriage body | svod/arch |
| lavanda/lavender | svecha/candle | kvartira/apartment | taliya/waist |
| lopata/shovel | svistok/whistle | ladon'/palm | teatr/theater |
| lyul'ka/cradle | telefon/phone | lak/varnish | trassa/highway |
| mashina/car | tigr/tiger | luzha/puddle | trotuar/pavement |
| mina/mine | topor/axe | lyazhka/thigh | ugol/corner |
| nozh/knife | ukho/ear | maslo/butter | ulybka/smile |
| nozhnitsy/sciccors | ulitka/snail | matryoshka/nesting doll | ushchel'ye/ravine |
| odekolon/cologne | utka/snail | mollyusk/shellfish | veranda/veranda |
| ogon'/fire | utyug/iron | nomer/number | veyer/fan |
| orkestr/orchestra | veter/wind | ostrov/island | vino/wine |
| pal'ma/pal'ma | volchok/gyroscope | paket/package | yarmarka/fair |
| parikmakher/hairdresser | vrach/doctor | park/park | yubka/skirt |
| pevets/singer | zerkalo/mirror | pasport/passport | zaliv/bay |
| zoloto/gold | | zoopark/zoo | |
